# A qualitative evaluation of Southwark Council’s public health response to mitigating the mental health impact of the 2017 London bridge and borough market terror attack

**DOI:** 10.1186/s12889-021-11447-8

**Published:** 2021-07-19

**Authors:** Sandra Jumbe, Adrienne Milner, Megan Clinch, Jonathan Kennedy, Richard J. Pinder, Carolyn A. Sharpe, Kevin Fenton

**Affiliations:** 1 Institute of Population Health Sciences, Queen Mary University of London, Yvonne Carter Building, 58 Turner Street, London, E1 2AB UK; 2 College of Health, Medicine Life Sciences, Brunel University London, Heinz Wolff Building 210, Uxbridge, UB8 3PH UK; 3 Department of Primary Care and Public Health, School of Public Health, Imperial College London, Reynolds Building, St Dunstans Road, London, W6 8RP UK; 4 Southwark Council, 160 Tooley St, London, SE1 2QH UK; 5 Public Health England, Fleetbank House 2-6 Salisbury Square, London, EC4Y 8AE UK

**Keywords:** Terror attack, Mental health, Qualitative research, Public health

## Abstract

**Background:**

Over recent years there have been several major terror attacks in cities across Europe. These attacks result in deaths, physical injuries, and pose long-term threats to mental health and wellbeing of large populations. Although psychologists have completed important work on mental health responses to disaster exposure including terrorist attacks, the mental health impacts of such attacks have been comparatively less examined in academic literature than the acute health response to physical injuries. This paper reflects on Southwark Council’s pioneering public mental health response to the June 2017 terror attack at London Bridge and Borough Market. It aims to explore perceptions of the mental health impact of the incident by those living and working in the borough.

**Methods:**

A rapid qualitative evaluation informed by the logic underpinning Southwark Council’s response was conducted. Seven formative interviews were undertaken with individuals involved in the response planning and/or delivery, enabling the evaluation team to establish the response’s theoretical basis. Subsequently, nineteen semi-structured interviews with consenting Council employees, residents, business owners, and workers from the Borough were conducted to understand perceived mental health impacts of the attack and the success of the Council response. Thematic analysis of transcribed interviews was undertaken to evaluate the extent to which the response was implemented successfully.

**Results:**

Participants reported feeling the attack had a wide-reaching negative impact on the mental health of residents, those working in the borough and visitors who witnessed the attack. Delivering the response was a challenge and response visibility within the community was limited. Participants suggested a comprehensive systematic approach to health needs assessment informed by knowledge and relationships of key Council workers and community stakeholders is imperative when responding to terrorist incidents. Improved communication and working relationships between statutory organisations and community stakeholders would ensure community groups are better supported. Prioritising mental health needs of terror attack responders to mitigate persisting negative impacts was highlighted.

**Conclusions:**

This article highlights a potential public health approach and need for developing robust practical guidance in the aftermath of terror attacks. This approach has already influenced the response to the Christchurch mosque shooting in 2019.

## Background

Over recent years there have been a number of violent attacks that have targeted civilians in major cities in Europe and elsewhere. The most notable of these include the November 2015 Paris (France) attacks (including the Bataclan Theatre) that resulted in 130 deaths, the truck attack in Nice (France) in July 2016 which left 87 people dead, the Manchester Arena (United Kingdom) bombing that killed 23 in May 2017, and the Christchurch (New Zealand) shootings that led to 51 fatalities [1–3, 14]. The immediate physical harm caused by terror attacks are managed by emergency blue-light services using well-established protocols. However, such incidents also have longer term impacts on the mental health and wellbeing of a much larger population [1]. Indeed, it is through this sense of long-term anxiety that terror targets undermine the mental health and wellbeing of the target population. Despite being widespread and long lasting, these effects are less tangible and protocols to deal with them are less well-established since they have attracted comparatively little academic research [2]. It is crucial that learning is derived from responding to terrorist attacks and other major incidents to improve future management of such events [2, 3]. This article examines the innovative approach pioneered by Southwark Council’s Public Health team who sought to characterise and mitigate the mental health and wellbeing effects of the London Bridge and Borough Market terror attack that occurred on 3 June 2017 [4].

The incident took place at ten o’clock on a Saturday evening amid the busy bars, restaurants and nightlife found adjacent to the River Thames in the centre of London, United Kingdom. The attackers drove their van into pedestrians on London Bridge before the three attackers proceeded to the Borough Market area on foot, where they attacked people with knives. Eight members of the public were killed, and the three attackers shot dead by London’s Metropolitan Police. A further forty-eight people received hospital treatment for physical injuries, twenty-one of these in critical condition [5].

In addition to the fatalities and direct physical injuries, there were approximately 850 witnesses and between 1000 and 3000 people were evacuated from the area. Following the attack, police cordoned off Borough Market and the surrounding streets. Two hundred and twenty-eight businesses were in the initial wider police cordon and 50 in the inner cordon. Much of the cordon was lifted on Wednesday 7 June but the Borough Market area (home to many market stalls, eateries and drinking venues) did not re-open until Wednesday 14 June – 11 days after the attack. Beyond these tangible consequences, it is clear that the attacks caused considerable distress, characterised by psychopathology symptoms and unpleasant emotions (but not classified as a psychiatric disorder) to the population in Southwark and beyond [1, 3]. The leader of Southwark Council wrote that this was “an attack on the diversity we celebrate - the mix of nationalities drawn to our borough – and the great pleasure we take in our restaurants, bars and markets” [6].

### Evidence base

Research into the mental health impacts of terror attacks shows they are difficult to quantify [3]. Factors including proximity to the attack, previous mental health issues, heightened media use, and previous exposure may cause more adverse outcomes related to post-traumatic stress disorder (PTSD), post-traumatic stress symptoms (PTSS), prolonged grief disorder (PGD), and insomnia [7–12]. Those trained to respond to a terror attack, as well as those with certain personality dispositions, are less likely to experience mental health consequences following an incident. Those who have previously experienced a terror attack, been highly exposed to subsequent media coverage, and volunteers with no professional rescue training or experience are more likely to suffer negative mental health consequences such as PTSD and PTSS [13, 14]. Empirically confirmed successful mental health interventions include preparedness programmes, cognitive behaviour therapy, exposure narratives, and eye movement desensitization and reprocessing [15–17]. The evidence base for the effectiveness of psychological first aid as a post-disaster intervention remains inconclusive [15].

Research into psychosocial outcomes indicates that the clinical presentations of PTSD following terror attacks are often complex [1, 4]. There may be long-term negative impacts on survivors, their family members and at times the wider community. Work, education, and personal and social lives are often negatively impacted [18, 19]. Generalised trust of other people may also be affected [20]. Involvement in community activities may buffer adverse psychosocial outcomes [21].

Research on individual- and community-level outcomes demonstrates that an individual’s behaviour to others may become more altruistic but individuals and community economic potential suffers [22–25]. There are also demographic differences related to gender, race, and religion in individuals’ responses to terrorism that should not be ignored when seeking to understand and respond to such events [26–28]. Research on terror attacks and community resilience suggests that stakeholders should promote community resilience because it is linked to a wide range of positive outcomes, as well as being protective against negative outcomes for communities and individuals [29–34]. Finally, there is also evidence that overzealous mental health and wellbeing responses in terms of reviewing trauma in detail and at length on the same day it is experienced may be disadvantageous in relation to psychological health outcomes [35].

This inconclusive review of the published evidence demonstrates the need for more research, emphasised by the important knowledge gap surrounding how public health professional may work to mitigate mental health and wellbeing impacts of terror attacks, as opposed to humanitarian emergencies more generally [2]. This review also suggests Southwark Council were delivering an innovative and challenging programme of work very rapidly in the context of very limited evidence and guidance.

This paper reports findings from a commissioned rapid qualitative evaluation which aimed to: 1) understand how this terror attack impacted on the mental health and wellbeing of Southwark Council community and the process of implementing the response; 2) describe and explore the usefulness/value of Southwark Council’s public health response; and 3) contribute to the body of knowledge on this topic to inform development of best practice guidance for local government organisations who might face similar situations.

## Methods

### Design

We conducted a qualitative evaluation of Southwark Council’s public health response to the London Bridge terror attack, within the London Borough of Southwark. This evaluation, commissioned over a 3-month period, involved two phases. Firstly, formative semi-structured interviews with key informants who were Southwark Council employees or statutory service employees involved in the response to gain an in-depth understanding of the response, how and why it was delivered. Phase two involved interviews with adults from the Borough’s community i.e., local business leaders, their employees or Southwark Council residents, to explore their views of the mental health impact of the attack, the value of the response and the process of implementing the response. Evaluating the impact of the response on the mental health of those who received it was outside the scope of the evaluation.

### Sampling and recruitment

This study received ethical approval from the Queen Mary Ethics of Research Committee on 23 May 2018 [QMERC2018/35]. Following this, four researchers (one male; three females) from Queen Mary University of London (QMUL) approached individuals by email or telephone about the commissioned study.

**Fig. 1:**
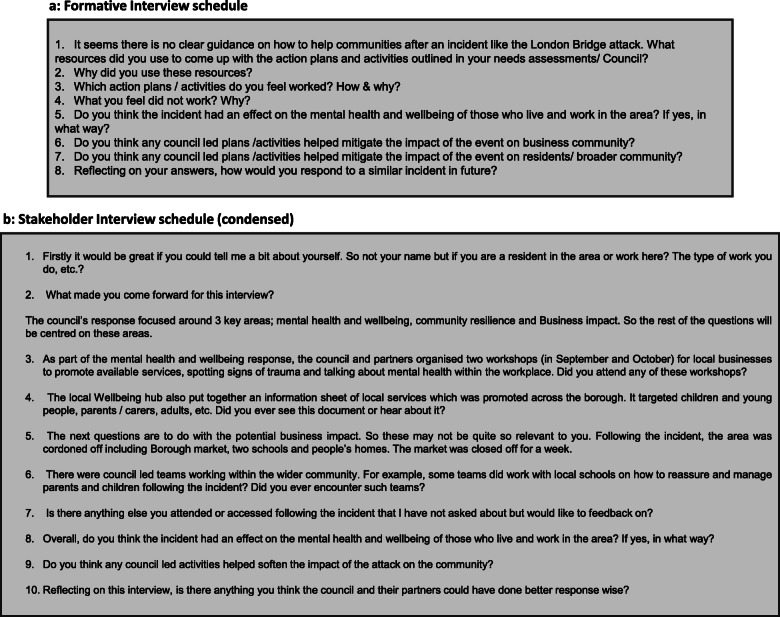
**A** Formative Inteview schedulel. **B** Stakeholder Interview schedule (condensed)

The first seven interviews were formative in nature and were conducted with Southwark Council employees directly involved in the planning and/or delivery of the mental health and wellbeing response (see Fig. 1A). The aim of these interviews was to develop an understanding of aims of the response, the appropriateness and success of which would then be explored in further semi-structured interviews with people who lived or worked in the Borough. There was no relationship between researchers and these participants prior to the research and the participants were all identified by Southwark Council as individuals who were deeply involved in delivering the mental health response. QMUL researchers contacted the potential interviewees via email, explaining the project and asking if they wanted to participate, while making it clear that they were not obliged to do so. Participants were informed both in the participant information sheet and verbally by QMUL researchers that the study purpose was to understand the strengths and weaknesses of the Council’s public mental health response to the terror attack. We hoped to achieve this by interviewing a variety of people who implemented or were exposed to the response. These interviews, combined with an analysis of response documentation acquired from Southwark Council’s Public Health Team by AM and JK, informed an understanding of the evidence and logic underpinning the Council’s response activities and the conditions under which it was developed. They also informed the design of the interview protocol for the further interviews with local residents’ community leaders, business owners and workers.

After this formative evaluation stage, 25 local residents, workers, businesspeople and community members were then approached for interview. Those willing to participate responded to provide written informed consent and arrange an interview with researchers at a date and time of their convenience. Nineteen out of the twenty-five individuals approached agreed to participate. Out of the six who did not participate, three did not respond to the email and one was on annual leave. The other two declined because they were too busy with organising the incident’s one year anniversary activities, which was near the time the interviewes were conducted.

As mentioned previously, understanding garnered from the seven formative interviews informed the questions used in interviews conducted with members of the local community (local business owners, Southwark Council employees, Southwark residents and community leaders, head teachers, and police officers) who had lived experiences of the terror attack (see Fig. 1B). In these interviews they provided their experiences living through the terror attack, and insights into how the incident impacted the mental health and wellbeing of their community. Furthermore, because part of Southwark Council’s response to the crisis included attempting to understand and meet the mental health needs of the community, participants in the second round of interviews provided their views on how visible, effective or helpful the Council’s response was. It should be noted that the aim of these interviews was not to ascertain the effectiveness of the response; instead they aimed to explore the extent to which the logic underpinning the response related to the experiences and perceptions of the participants and their views on how it was implemented.

Purposive sampling aimed at recruiting a diverse range of people for the subsequent interviews was used to obtain wide ranging narratives from the community [36]. There were no incentives or compensation for study participation. Key characteristics of the sample are presented in Table 1.

**Table 1:** Demographic characteristics of participants (*n* = 19)

Characteristic		Value (n(%))
Gender	Male	11 (58%)
	Female	8 (42%)
Ethnicity	White	16 (84%)
	Non-white	3 (16%)
Southwark Council employee		5 (26%)
Other i.e. police service, local mental health teams, faith leaders, headteachers, business owners, residents)		14 (74%)

### Data collection

All but three interviews were conducted face to face, either at the participant’s workplace or alternative community venues between 23 May and July 2018. The other three interviews were done over the phone. The interviews took approximately 45 min. All interviews were audiotaped, professionally transcribed and deidentified using pseudonyms to protect participants’ confidentiality. Researchers wrote field notes after each interview to record contextual data and aid reflexive thinking. Data collection and analysis occurred simultaneously. As data collection proceeded, interview transcripts were reread and analysed by SJ and MC using a thematic analysis approach. This simultaneous approach allowed the researchers to effectively identify when data saturation was reached, and data collection should stop. Data collection halted at 25 interviews (7 formative, 19 summative).

### Data analysis

Two researchers (SJ and MC) used inductive thematic analysis to analyse the seven formative interview transcripts and identify emerging themes. They independently coded the transcripts line by line and subsequently met on two occasions to compare findings alongside the corresponding rationale that they independently identified whilst reviewing the interview transcripts, and to discuss any differences in their codes and (sub)categories. SJ and MC used the seven formative transcripts to each develop a codebook containing code names, descriptions, categories and subcategories with quotations that illustrated each aforementioned element. These codebooks were used to present findings that emerged from the formative interviews to the wider team (AM and JK) during a weekly team meeting, to inform recruitment strategies and subsequent interview schedules for the summative interviews. Weekly meetings also resulted in the refinement and addition of new codes to the coding frame. A similar iterative and collaborative analytical approach was taken for the summative interviews. SJ and MC independently coded transcripts and identified emerging themes. The coding and relationships between themes were further discussed with the wider study team and revised accordingly when consensus on discrepancies between the coders (SJ and MC) was met. Through this strategy common themes related to the perceived mental health impact of the incident and the visibility and appropriateness of the Council’s mental health response were ascertained.

The interviews were conducted by qualified researchers. The core research team composed of a psychologist and three senior global public health lecturers (a medical anthropologist and two sociologists) with combined expertise in qualitative research, evaluative methods and public health interventions development. From a contextual perspective, the team’s collective research interests in the areas of mental health and public health, including the impact of violence on health and health inequalities drew them to conduct this commissioned qualitative evaluation. The core team (SJ, AM, MC, and JC) had no prior relationship with Southwark Council or the community before this work. However, their pre-existing relationships from doing community engaged research with North East London local groups further motivated the team to do this important work. Having conducted numerous research and evaluation frameworks for several local authorities, MC was familiar with delivering commissioned work within these settings. Being London based meant the team benefitted from local geographical knowledge which made it easier for them to be flexible with when and where participant interviews could take place. The team’s varied topical and qualitative methodological expertise resulted in a range of interpretations and reflection on the data through a variety of perspectives. The use of such a structured approach enabled the team to conduct a rapid and focussed analysis [37].

### Patient and public involvement

Due to the sensitive nature of this work, the researchers worked closely with key community leaders and informants from Southwark Council to devise an appropriate data collection method, particularly the interview questions. Southwark’s Public Health division continues to work with local community groups to help disseminate and comment on our findings at relevant community events.

## Results

**Fig. 2:**
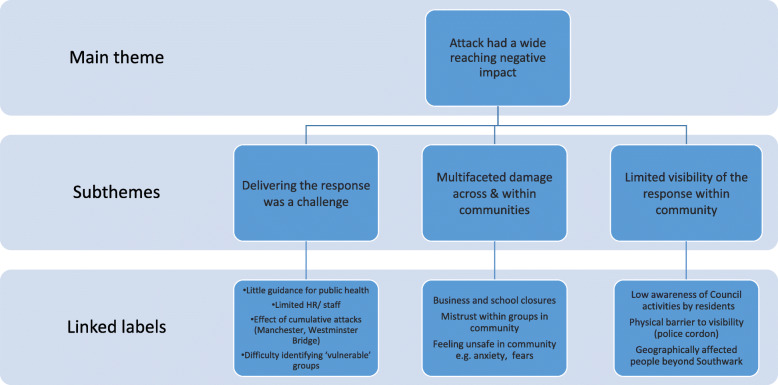
Thematic map

Themes that emerged from participants’ narratives were merged into overarching categories in Fig. 2, to describe key findings within the data. Table 2 contains anonymised quotes from participants to further support findings and illuminate their experiences.

**Table 2:** Verbatim quotes from study participants

Quote number	Quote
1	I from the outset was quite concerned about the welfare of everyone who had been affected by this. Local residents, Borough Market, the market stall holders. Folk who had witnessed this, folk who had been injured by it. I knew there was a strong international dimension from the get-go and I wanted a safe refuge for family members coming from abroad to be able to go close to the site. To have good psychological support, good practical support. So we opened a Humanitarian Assistance Centre. (Council worker)
2	We put in place also like an information sheet which was promoted across the borough that had services for children and young people, for parents, carers, for adults. But the workshops that people can access... so the idea was about developing toolkits, linking people with support which is in the borough, and having that seminar to understand more what is out there. Taking that back to there, what is within the area. (Local mental health team)
3	We used the council’s CCTV network to provide cover to faith institutions, because this was happening during Ramadan where there’s a large amount of people out on the streets in the evening having come to and from Friday prayers in particular. They (Muslim community) were worried about attacks against their premises, they were worried about hate crime and victimisation of their communities. (Council worker)
4	Imagining the kind of psychological stuff, of imagining what could’ve happened to you. There’s the people who work here in the restaurants and everything. I mean some of them saw the most horrific things and felt that their life was on the edge as they were cowering at the back of their restaurants. And some staff had just gone because they couldn’t cope being in the place anymore. (Community leader)
5	there were photographs in the press, where people actually have obviously used a long lens and were outside the court and from someone’s bedroom shooting down the road (resident)
6	we were already being asked to do interviews. I say we, the authority and the leader, that kind of level to do interviews. The news is straight on it, ain’t they? (Council worker)
7	When it comes to the women, because they use a hijab, it’s very obvious. But men, like me now, I carry my cap and put it in my pocket, nobody will know. And when it comes to having a beard, not only Muslims have a beard. We have some non-Muslims that they just like the beard for fashion. You understand it? So when it comes to the women they are more vulnerable (Muslim community leader)
8	Why were we locked out of our … either out or into our homes? And why was the cathedral locked down? People couldn’t access the sacred place that’s in the middle of the community. So people were being told you can get in and turn up and told no, you can’t. It causes frustration, it causes angst and causes just anger. I was awake all night really and discovered that the cordon was Pizza Express on our side of Bankside. And the officers there were very clear that I wouldn’t be able to get to the cathedral. (Resident)
9	I think the people who attended [the workshops] were mostly businesses who attended. I think it would be useful for people who were actually affected from the event, the incident, to allow them to come. (local mental health team)
10	The question I always get is how do we identify ‘vulnerables’? And we always kick back with well what’s vulnerable? Because three o’clock in the morning, you’re chucked out on the street, you’re all vulnerable. (Council worker)
11	People who had gathered there spontaneously started to clear all the flowers after the mayor and the clergy and some people from the mosque had begun it, everybody then joined in. And everyone cleared it themselves, the community cleared the flowers, which was extremely cathartic. The council workers who were there to put everything into the vans, just stood there and received all the bunches from the people and put them into the vans. And that was really, really good. (Community leader)

### The public health response

Formative interviewees reported that a Humanitarian Assistance Steering Group (HASG) was convened by Southwark Council in the immediate aftermath of the incident. The HASG brought together multiple stakeholders from the borough and wider region to develop a humanitarian response. An early, and unusual, decision of the HASG was to set up a Mental Health and Wellbeing Sub-Group (MHWSG) co-chaired by the local Director of Public Health and the chief executive of the local mental healthcare organization.

At the direction of the Director of Public Health, in the days immediately following the incident, the public health team undertook in parallel a rapid literature review which drew on expert opinion and experience from the recent Manchester Arena attacks, and a health needs assessment. Their review identified limited and contradictory guidance regarding best practice in such situations. In the most important response frameworks, terror attacks are treated as a humanitarian emergency that are indistinguishable from natural disasters. Although exposure to natural disasters also increases risk of adverse mental and behavioural outcomes, this overlooks the fact that terror attacks *sui generis* aim to cause long-term harm to mental health and wellbeing of communities.

The health needs assessment drew on opinion and perspectives from around the Council, wider stakeholders and the community. The circumstances meant that the needs assessment itself required a dynamic approach with the Council’s public health team describing it as a “live document”. Three principal areas of interest were identified: mental health and wellbeing of those affected by the incident (with varying degrees of direct or indirect involvement), economic activity (that is the businesses affected), and broader community cohesion.

Needs were identified within hours of beginning the needs assessment. In the absence of any existing guidance, Southwark Council’s Public Health team coordinated a variety of initiatives aimed at protecting and promoting the mental health and wellbeing of people affected by the attack (quote 1). An early and very practical need was to review the evidence around risk communication so that social media messaging could be safe and effective. A compendium of social media messages drawing on the evidence base was pulled together.

Other activities included signposting people to pre-existing services and producing a wellbeing factsheet. They also arranged mental health workshops that were positively received by attendees from the local community and businesses (quote 2). From our evaluation there is evidence that the techniques used in these workshops had a broader positive impact. For example, a local schoolteacher used some of the techniques taught at the workshop with her pupils.

The MHWSG oversaw the design and response of all public health activities, whilst providing an interface with the NHS and other community partners such as business leaders and faith groups. This sub-group met approximately weekly for the first month and then on a reduced frequency, thereafter, advocating upwards to the HASG for parity between physical and mental health response. It also laid the groundwork for the subsequent Outreach and Screen Programme, deploying trauma-focused cognitive behavioural therapy and other interventions. The MHWSG stood down almost four months after the incident. However, the Director of Public Health continued to provide advice and leadership around mental health and wellbeing within the HASG for the following two years, until that group was stood down.

Southwark Council also made efforts to protect potentially vulnerable communities at a time when police resources were stretched. For example, the Council supported and reassured the Muslim community by providing CCTV surveillance around mosques at their request (quote 3).

### Perceived impact on mental health and wellbeing

Interviewees highlighted numerous ways in which the London Bridge and Borough Market attack negatively impacted the mental health and wellbeing of people living and/or working in the area. Overall, the attacks resulted in a general sense of sadness, anxiety and fear in the local community (quote 4).

A number of contextual issues were raised. Firstly, the attacks took place in an area where lots of people from all over the city – and the world – come to socialise. The fact that it occurred near a major central London railway terminus appeared to have exacerbated the feeling that “it could have been me”. Conversely, it also meant that the impacts extended well beyond the geographical confines of the borough of Southwark. For example, none of the dead were residing within Southwark. Reflecting the global nature of London, only one of the eight victims and one of the three attackers were British nationals. Secondly, the London Bridge and Borough Market attacks came very soon after several other terror incidents in London (Westminster Bridge attack, three months prior) and the UK (Manchester Arena bombing a fortnight prior). It is possible, if not likely, that the cumulative effect of these attacks led to a heightened sense of anxiety and fear in the general population, as well as placing additional pressure on response resources. Third, this particular incident received a great deal of media attention because it occurred in a location proximal to many UK media offices and in the middle of a global city. Council employees and partners reported that intrusive press coverage impacted on their ability to respond to the attack (quotes 5 & 6).

Interviews were conducted around the first anniversary of the attack and negative impacts on this community’s mental health and wellbeing were still apparent. For example, several workers in Borough Market did not attend work on 3 June 2018 so they were not reminded of the incident.

Minority groups appeared particularly negatively affected. As the attackers had referred to Islam in order to legitimise their actions, the local Muslim community was fearful that they might become a more salient target of hate crimes. A local Muslim community leader expressed concern that female Muslims were especially vulnerable because their hijabs made them easily identifiable (quote 7). Two factors heightened the fears of the local Muslim community. First, the attack occurred during Ramadan, when the community would gather together far more often than usual. Second, there were a number of recent terror attacks carried out by British Muslims who had been inspired by the so-called Islamic State (in Syria and Iraq) and came less than a month after the Manchester Arena attack. However, it was noted that the longstanding and strong relationship between the Muslim community and other faith communities in Southwark did help quell these concerns and issues of community cohesion.

The impact of the police cordon on local residents and businesses was another regularly reported issue. In the aftermath of the attack, the needs of the police to secure a sterile crime scene took precedence over the mental health and wellbeing needs of the local population who were deprived of their accommodation, sustenance, income and systematic mental health support delivered by experts.

Local people who lived within the cordon were evacuated from their homes and prevented from returning while the investigations continued. In some cases, it was ten days before people were permitted to return home. At a time when many residents were already feeling anxious and upset, this uncertainty created additional stress (e.g. not being able to feed pets or access possessions needed for work). Several interviewees explained that this issue was worsened by a perceived lack of information from law enforcement. Even a year after the incident, resentment towards the police among local communities was observed (quote 8).

The closing down of Borough Market resulted in a negative economic impact on a variety of people in the area. Business owners reported they had lost “vast amounts of money” as they were prohibited from opening for up to ten days, including two weekends. This was particularly problematic for small business owners (who make up a large proportion of businesses in Borough Market). This problem was most apparent among market traders and others who held fresh foods in stock, as it perished while the cordon was up. Staff working within the cordon were also affected: with no workplace, some had no income.

### Was logic underpinning the response, and its implementation, appropriate?

Interviewees acknowledged that the Council was operating under extreme circumstances amid very little guidance. Moreover, they had limited human resources and members of the teams deployed had many other roles and responsibilities. For example, the Council’s Emergency Planning team which has statutory responsibility to undertake specific tasks under the Civil Contingencies Act 2004, comprises only three staff members. Like many other council departments, Southwark’s Public Health team re-tasked six members of staff immediately into supporting the response. There was also an array of other unexpected tasks that arose, for example providing information and reassurance to local community stakeholders.

There was an issue with lack of visibility of the response. Only a limited number of residents were aware of or accessed the Council signposted mental health and wellbeing initiatives. Most stakeholder interviewees said they did not attend the mental health workshops (quote 9). Many stakeholders reported hearing about the wellbeing information sheet, but very few actually saw it. Potential reasons for the low visibility include the physical barrier of the police cordon, staff not living in the area, and the difficulty in identifying those affected due to the transient nature of where the attack occurred.

Interviewees involved in the mental health outreach activity highlighted the challenge of contacting affected residents. Council workers did not have an immediately clear understanding of who was displaced and who had been affected. As a result, support ended up targeting the business community (specifically the Market) rather than residents (quote 9). A significant proportion of people present at London Bridge and in Borough Market on the night of the attack were not from the local area and this was a particularly challenging and heterogeneous community to target. This made it difficult for Southwark Council to trace individuals who required support and provide it in an appropriate manner (quote 10). Another reason for this lack of visibility is potentially tied to the ‘watchful waiting’ approach suggested by the evidence on trauma used by the MHWSG. Watchful waiting in the context of trauma exposure involves carefully monitoring people’s symptoms to see whether they improve or get worse. It is sometimes recommended because most people who develop problems after a traumatic experience get better within a few weeks without treatment [37]. This approach was sometimes misconstrued as the absence of a response by the community. In the case of Southwark Council, the watchful waiting service was commissioned in October (post-incident), meaning only a proportion of the exposed population were screened. Despite NICE guidelines for PTSD [38] stating the importance of screening post major event, in reality the national health service *‘do not run a continuous service, the event takes place, then they are making a decision if they want the service or not. And then it takes a few months to book someone onto the service’*. This limits ability for such commissioned services to effectively screen and monitor populations effectively.

Yet there were also several positively received measures that the Council took as part of their response. They appreciated the value and need for a bottom-up approach to encouraging community cohesion based on the social capital and relationships already embedded in the local community. One particularly positively observed outcome was a series of organically developed events, led by the community and supported by the Council, which brought a diverse range of people together to reflect on the incident. A ceremonial clearing up of flowers was a notable example of this (quote 11). The Mayor and local religious leaders began to move the flowers and other participants at the event and formed a human chain to help. The Council were at the end of the chain to respectfully place the flowers into a van. This was interpreted by many as an act that allowed the process of moving these offerings in a respectful manner. It is critical to recognise that such events were meaningful because they were driven by a number of community leaders working together in a proactive way to bring their local populations together.

Some members of Southwark Council had longstanding and trusting relationships with various community groups and were sources of reassurance and practical support after the attack. However, such relationships were not systematically engaged to enrich the response. For example, the relationships with representative from the local Muslim community fostered by various Council departments were not drawn upon to maintain and nurture community cohesion between different religious groups who lived and worked in the Borough. These stakeholder narratives suggest that local authorities should focus on building resilience, particularly among vulnerable populations to mitigate subsequent terror incidents. When incidents do happen, they should draw on such networks of resilience when planning and delivering a response. These same networks of resilience could also function as a means of systematically and sensitively disseminating information (i.e., the mental health and wellbeing leaflet) to people in need.

Finally, as some interviewees pointed out, organic community-based responses to the attacks did result in the protection of wellbeing as they forged strong relationships between people who were affected by the harrowing and unique event. Again, it is important to think about how such relationships might be nurtured in the event of future responses.

## Discussion

The attack was reported to have had a profound and negative impact on the mental health and wellbeing of those we interviewed and those citizens for which they felt a responsibility for caring for in a variety of ways similar to previous terror attacks [1, 7, 8, 19]. General consequences included the shock and trauma of being in close proximity to a terror attack. Context specific consequences included the distress caused by businesses having to cease trading because of the police cordon, and the economic impact this would have on business owners and their employees. Some of these consequences were general and some were context specific. Some impacts were in direct response to the terror attack while others were borne out of the post-incident actions related to the criminal investigation, which left the needs of residents and business unmet [39]. For example, the police impose a cordon on the area in order to carry out the criminal investigation. This resulted in residents being locked out of their homes for days – and not being able to access pets, clean clothes, and so on – and businesses being unable to trade, so the many restaurants and stall around Borough Market were left with decomposing produce that they could not sell.

Crucially, there is limited literature from a public health governance on how to mitigate mental health and wellbeing impacts of terror attacks, as opposed to humanitarian emergencies more generally [2]. This is an important knowledge gap given the context of terror seeking to stoke population-level anxiety. In spite of this gap, Southwark Council used the scant evidence available and responded to the attack with a pioneering public health approach [4]. By developing a Health and Wellbeing Sub-Group and deploying a dynamic health needs assessment-based approach, Southwark’s Public Health team were able to capture and respond to a range of emergent needs in real-time and exploiting the evidence-base where it was available. This approach has highlighted a more strategic gap in emergency response around ensuring a parity of esteem between physical and mental health. This paper is important because it enables us to learn lessons from this approach and inform the development of guidelines for responses to future terror attacks that take place in similar urban environments. Since the 2017 attacks, the Association of Directors of Public Health has drawn on Southwark’s experience to issue new guidance describing a role for public health in the acute and longer-term response. This evaluation shows that people used signposted support services and workshops, and that Council activities mitigated some of the negative impacts on mental health and wellbeing driven by the terror attack.

There are important lessons that can be learned from this evaluation. First, the value of using pre-existing social relationships to enhance reach of public health efforts is critical. Pre-existing community cohesion also allows people on the ground to come together and help each other practically and emotionally with the Council’s support [17]. Second, there is a serious question posed in how to better balance the needs of criminal justice process with the mental health and wellbeing of relevant communities. More research is needed to understand how these processes can be better aligned and implemented alongside public health priorities in emergency situations without undermining one another. Third, identifying people in need was a challenge, exacerbated by the incident affecting a high heterogeneous and mobile population amid a global city. In the future, and perhaps in partnership with locally elected politicians and health services, the municipal government should re-double the efforts to analyse and understand their geographical population more systematically to better identify people’s needs, particularly within vulnerable groups. This would facilitate more focused and targeted interventions based on the needs of the community in terms of demographics and culture. Fourth, additional work is also needed on addressing the wellbeing needs of Council staff and other partners who may or may not be involved in the initial ‘blue-light’ response [40, 41]. Workers such as those cleansing the urban environment from victims’ blood or repairing bullet-holes in walls are sometimes overlooked in the immediate aftermath of such an incident. This is particularly important when resources are overstretched. This group of workers may also relate more closely to the environment: working there in the future unlike emergency workers drawn from a larger area who may have only a fleeting relationship with the incident zone.

Overall, four areas of learning for local government public health responses to terror attacks have been identified in the evaluation:

1. It is necessary to undertake systematic and appropriate identification of community mental health and wellbeing needs at all stages of the incident response. This process should build on pre-existing local intelligence and involve a robust and practical monitoring and evaluation process.
2. Strong communication between emergency services and the Council, specifically with regard to an awareness and management of the social and mental health effects of their work, needs to be enabled.
3. Collaboration and communication with pre-existing health service mental health and other services (social care, education, housing) teams is crucial in the targeted delivery of a mental health and wellbeing response.
4. Strategies for monitoring and supporting the mental health of council workers involved in recovery activity must be embedded in local authority responses to terrorist incidents.

Our study has several strengths including a rigorous qualitative design, successfully engaging with key community leaders from a participant population that is historically underrepresented in research (Muslim community), and the generation of rich descriptions of both how the Council’s public health response was delivered and the intervention’s perceived reach within the community. By providing feedback about areas of significance, this research will help in planning for future acts of terrorism and/or disasters.

Study limitations include the short 3 month timescale of the commissioned work. Three out of the 25 potential participants did not respond to the study invitation email and we were unable to follow them up beyond the timelines of the commissioned work. These individuals worked in the schools and mental health services so we may be missing additional perspectives on how the terror attack affected children and their families within the community or whether the attack changed working conditions for those in educational and/or health settings. Data saturation gave us confidence to stop interviewing but we do not know whether these three nonresponders could have provided additional perspectives beyond the data collected. Extending the recruitment period would have allowed us to interview additional participants and may have elicited additional insights. Some may argue that this research may also be limited by recall bias because we interviewed participants a year after the incident which may affect their recollection of events [42]. Recall is an issue for most research collecting retrospective data from a specific time point [43]. However, there was corroboration on specific events and timelines of activities from various participant accounts. For example, several participants described the impactful organic coming together of the community at a religious ceremony after the attack similarly. Moreover, considering the life changing experiences of those we interviewed, a terror attack especially one that took place only a year ago, is something hard to forget. We are therefore confident that recall bias was not an issue in our interview data [42].

## Conclusion

Terror attacks are likely to remain a continued risk for urban centres across the world. Despite governments’ best efforts to prevent attacks, increasingly a public health approach to mental health and wellbeing will be useful in mitigating the longer-term impact of terror. This evaluation demonstrates the value of evidence based-practice and public health leadership in the aftermath of a terror attack. Yet more research in this area is needed. We propose that future management of incidents of this nature will benefit from a similar approach that enshrines parity of esteem between mental and physical health, as well as acknowledging and mitigating the broader challenges to population-level wellbeing, economic risks and community cohesion.

## Data Availability

The lead author (SJ) affirms that the manuscript is an honest, accurate and transparent account of the study being reported. The datasets generated and/or analysed during the current study are not publicly available but can be made available from the corresponding author on reasonable request.

## Abbreviations

HASG: Humanitarian Assistance Steering Group
MHWSG: Mental Health and Wellbeing Sub-Group
NHS: National Health Service
CCTV: Closed-circuit television, also known as video surveillance
